# Short-Term Results of Operative Treatment of Primary Ileocecal Crohn’s Disease: Retrospective, Comparative Analysis between Early (Luminal) and Complicated Disease

**DOI:** 10.3390/jcm12072644

**Published:** 2023-04-01

**Authors:** Nicolas Avellaneda, Tora Haug, Mai-Britt Worm Ørntoft, Sanne Harsløf, Lars Peter Skovgaard Larsen, Anders Tøttrup

**Affiliations:** 1 Inflammatory Bowel Disease Section, Surgery Department, Aarhus University Hospital, 8200 Aarhus, Denmark; 2 Department of Surgery, Gødstrup Hospital, 7400 Herning, Denmark; 3 Diagnostic Imaging Department, Aarhus University Hospital, 8200 Aarhus, Denmark

**Keywords:** Crohn’s disease, surgery, colectomy, postoperative complications

## Abstract

Early surgical treatment for patients with ileocecal Crohn’s disease (CD) could be an alternative to biological therapy. The aim of this study is to compare operative outcomes following ileocecal resection for patients with luminal and complicated CD. Patients operated for primary ileocecal CD during 8 years in one tertiary-referral hospital were allocated into 2 groups: those operated for early (luminal) disease (ECD), and for complications of CD (CCD). A retrospective comparative analysis was performed. A total of 273 patients were included in the analysis, 85 (31%) of which were in the ECD group. No difference was found regarding time from diagnosis to surgery. Surgical procedures were longer in the CCD group, with lower rates of laparoscopic approach (93 vs. 99%, *p* = 0.035) and higher conversion rates (20 vs. 2%, *p* < 0.001). ECD had non-significant differences in terms of major postoperative complications (9.4 vs. 14.9%, *p* = 0.215), shorter hospital stays, and lower rates of anastomotic leakage (3.5 vs. 6.8%, *p* = 0.285). Conversely, the CCD group had higher reoperation and re-hospitalization rates. Adequate timing for the indication of surgery in primary ileocecal CD, including an early discussion considering both medical and surgical treatment as options, could positively influence operative outcomes.

## 1. Introduction

Many patients diagnosed with Crohn’s disease (CD) present with a short segment of affected bowel limited to the ileocecal region [1]. For these patients, medical management with biological drugs is the current standard treatment after the failure of conventional therapy, and surgery is only indicated for patients who fail to respond to these drugs or present with complications of the disease [2,3].

Surgical treatment of patients presenting with CD complications is, however, associated with worse postoperative outcomes, including higher morbidity, requirements of perioperative optimization, and occasional stomas [4,5,6,7].

As an alternative to biological drugs, some studies have suggested that surgery for CD patients with luminal affection only, can provide better short- and long-term results [8,9,10]. Furthermore, this indication of surgery has been associated with improved postoperative outcomes, compared to surgery for complicated disease [6]. CD is well known to progress from luminal to complicated disease [1,11], and it is therefore justified to consider patients with luminal disease only as having “early disease” as seen in the context of the natural development of the disease.

Nevertheless, there is an ongoing debate related to whether this classification is adequate or not, which has been highlighted in a recent editorial by Pellino et al. [12].

The aim of this study was to assess operative outcomes of surgery for patients with primary ileocecal CD, comparing patients presenting only luminal disease at the time of surgery with those operated on for complications of the disease.

## 2. Materials and Methods

### 2.1. Ethical Considerations

The study was registered in the Central Denmark Region’s register of research projects (journal no. 1-16-02-200-22) and complies with the General Data Protection Regulation (EU GDPR) within the European Union.

Since the study is observational, retrospective, and without any intervention in patients’ usual treatment, ethics approval is not required according to Danish regulations.

### 2.2. Study Design and Setting

This study was conducted in a tertiary-referral inflammatory bowel disease (IBD) center in Denmark. According to Danish policy on centralization of patient care, this center receives and treats IBD patients from all over the Central Denmark Region (comprised of more than one million inhabitants).

The center has an experienced multidisciplinary team that focuses on IBD patients. This team is composed of physicians belonging to different specialties, including gastroenterology, surgery, internal medicine, diagnostic imaging, stoma nurses, nutritionists, etc. Additionally, specialized IBD surgeons, gastroenterologists, and radiologists meet once every second week to discuss patient management strategies of selected cases in an MDT setting.

### 2.3. Patient Management Strategy

To summarize the clinical management of patients with CD limited to the ileocecal region at this hospital, those with conventional treatment failure are initially stratified based on clinical and laboratory markers, colonoscopy, and high-resolution MRI, to diagnose likely fistulas or fibrotic strictures that leave patients ineligible for further medical treatment. After this, all cases are presented at the MDT conference. Here, the decision on whether to proceed with biological drugs or to offer surgery is discussed.

In patients that present with luminal involvement only, medical management versus surgery is discussed. The choice between second-line medication (biological agents) or surgery is based on both the individual MDT discussion and the patient’s personal preferences, after the patient has been informed by the gastroenterologist or IBD surgeon and received information about the advantages and disadvantages of each strategy.

For those who progress to second-line medical treatment, but later require a change in treatment (e.g., primary/secondary non-responders or adverse drug effects), the aforementioned process is repeated. As a result, a shared decision is made at each step before escalating to different lines of medical treatment.

**Figure A1:**
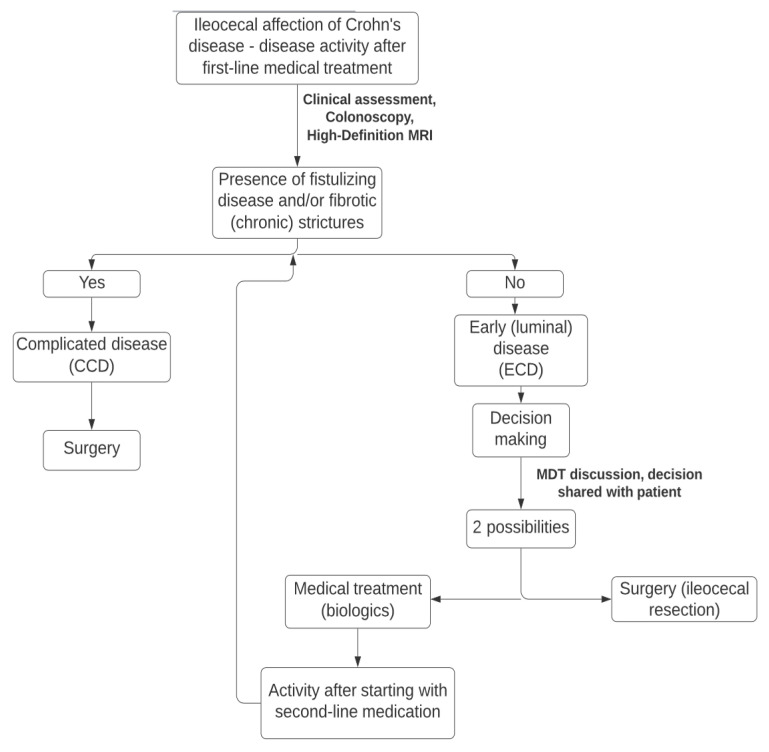
MRI features differentiating luminal and complicated disease.

**Figure A2:**
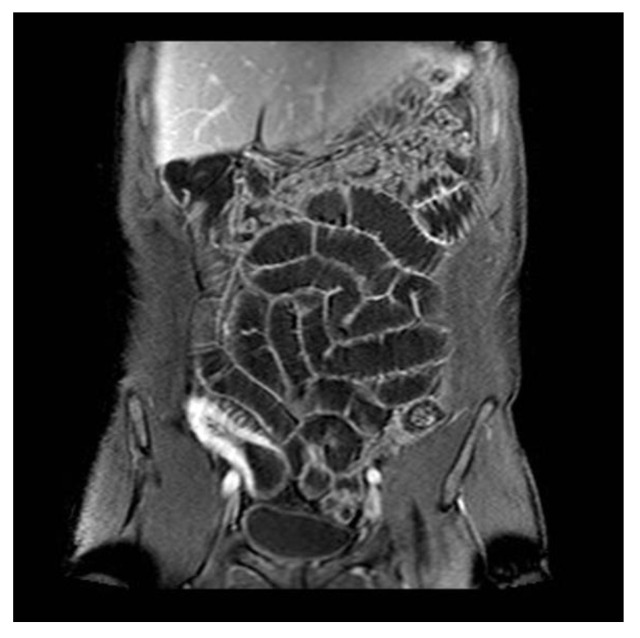
MRI visualization of a patient with luminal affection (Comb sign).

Appendix A shows a proposed algorithm for patients with ileocecal CD who have failed first-line medication and have luminal disease, as well as a summary of the most important MRI findings to differentiate luminal disease from fibrotic strictures (Figure A1 and Figure A2).

### 2.4. Inclusion and Exclusion Criteria

Patients submitted to primary resections for localized ileocecal CD (disease of the last 50 cm. of the terminal ileum and/or cecum) from 2013–2021 were included in this study.

Exclusion criteria were active tuberculosis, previous CD-related abdominal surgery, presence of CD activity in other intestinal segments at the time of surgery, or surgery indicated for causes other than CD.

**Figure 1:**
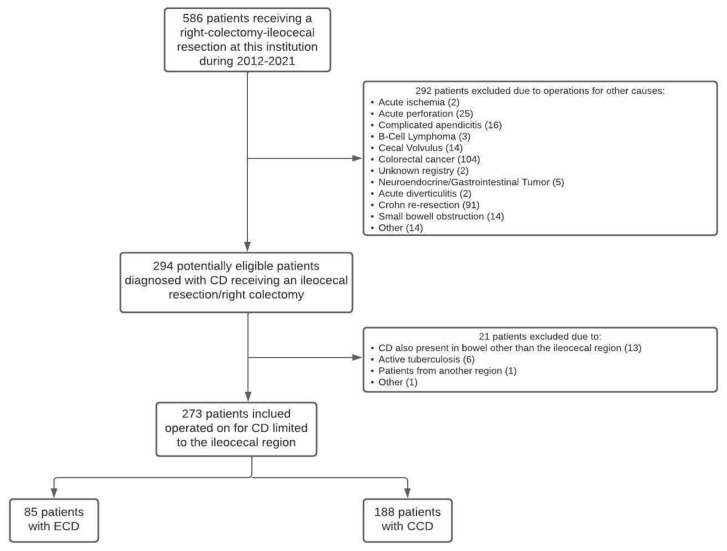
Patient selection process.

Figure 1 shows the patient selection process.

### 2.5. Data Collection and Management

A database was created for the purpose of this study using the RedCap platform (Research Electronic Data Capture, Vanderbilt University^®^), which complies with national and international regulations for data protection.

The variables included were chosen based on preoperative, intraoperative, and postoperative characteristics of CD patients considered relevant for the study’s purpose, and these can be found below.

After the principal investigator selected the variables, they were assessed by 3 consultants on colorectal surgery and 2 gastroenterology consultants specialized on IBD, to assess relevance.

Once the consultation process was finished, the database was filled in with patient information previously collected in a prospective way. The information of each patient was reviewed by 2 different people: a junior surgeon, and a senior colorectal surgeon.

For the data analysis, patients were allocated to 2 groups, according to whether they had been operated on in an early fashion (for luminal disease) (named Early Crohn’s Disease, ECD) or for complications of the disease (fibrotic strictures or fistulizing disease) (named Complicated Crohn’s Disease, CCD). As previously mentioned, the definition of early or late disease was made based on the disease phenotype, and not based on time from diagnosis to surgery.

To define the ECD group, we applied the Maruyama et al. definition [13]: Resection performed for inflammatory disease with purely luminal involvement, without previous resections (not related to postoperative recurrences), and no fibrotic stenosis or perforation which would make surgery mandatory.

### 2.6. Variables Analyzed

Information regarding comorbidities and previous exposure to medical treatment was gathered to assess differences between the two groups.

Preoperative variables: Charlson comorbidity score and WHO performance status, smoking (at the time of surgery), body mass index (BMI), presence of preoperative anemia, preoperative albumin levels, American Society of Anesthesiology (ASA) score, weight loss, and history of previous abdominal procedures.

Disease-related variables: Time from diagnosis of CD to surgery, Montreal classification of CD, preoperative exposure to biological agents within 12 weeks of surgery, perianal disease, exposure to chronic corticosteroids at the time of surgery (defined as having received more than 20 mg/day of prednisolone or equivalent for up to 6 weeks before surgery) [14], and requirement of preoperative nutritional optimization before the procedure (defined as patients who had to be hospitalized in order to receive enteral or parenteral nutrition before undergoing surgery).

Intraoperative variables: Operative time, character of the procedure (elective or emergency), operative approach and conversion rate, intraoperative complications (stratified according to CLASSIC Classification) [15], extension of mesentery resection (mesenteric sparing or wide resection as defined by Coffey et al.) [16], associated procedures (defined as an additional CD-related procedure other than the resection of the compromised bowel at the ileocecal region), requirement of more than one bowel resection, and performance of primary anastomosis.

Postoperative variables: Length of hospital stay, presence of major postoperative complications (Clavien–Dindo > 2) [17], fascial rupture, clinically significant gastrointestinal bleeding, abdominal abscess and surgical site infection (SSI), anastomotic leak rate (stratification based on the International Study Group for Rectal Cancer classification) [18], readmission and reoperation rates, as well as mortality within 30 days of the procedure. Finally, the length of the resected bowel was also assessed.

### 2.7. Outcomes

The primary outcome was to compare the 30-day overall major postoperative complication rate between ECD and CCD (Clavien–Dindo > 2).

Secondary outcomes were used to compare operative time, rate of laparoscopy, conversion, intraoperative complications, requirement of more than one bowel resection, the need for stoma, hospitalization time, reoperation, re-hospitalization, and mortality within 30 days of the original procedure.

### 2.8. Statistical Analysis

The Stata software was used for the analyses (v17, Statacorp, College Station, TX, USA). Categorical variables were described as percentages. Descriptive variables were tested for normal distribution using a Kolmogorov–Smirnov test. Normally distributed data were reported in means and interquartile ranges. Data that did not follow a normal distribution were reported in medians and ranges.

The Chi-square test and Fisher’s exact test (when appropriate) were used for the comparison of categorical variables, and the Student’s *t* test or the Mann–Whitney U were used for quantitative variables. Further on, the odds ratio (OR) with 95% CI were calculated.

A logistic regression model was used for multivariate analysis. The model included all the variables considered clinically significant by the authors. A *p*-value < 0.05 was considered statistically significant.

## 3. Results

A total of 273 patients met the inclusion criteria during the study period and were included in the analysis, 85 (31%) of which were in the ECD group and 188 (69%) of which were in the CCD group. The mean age for the whole group was 37 years (10–79), and 160 patients (59%) were female; the groups were homogeneous in terms of age and gender.

**Figure 2:**
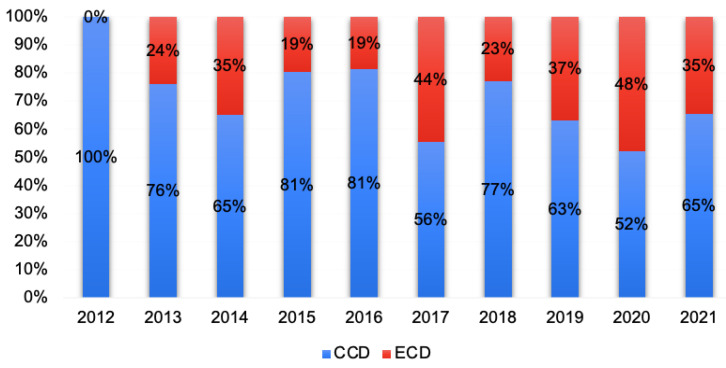
Distribution of indications for surgery per year, considering both groups.

Figure 2 shows the surgical indications for the patients included during the study period. A trend can be seen towards operating mainly on complicated patients during the first years, with an increase in indications for luminal disease during the most recent years.

### 3.1. Main Outcome

A total of 36 patients (13.19%) presented major postoperative complications during the 30-day follow-up. These events were more frequent in the CCD group, though this difference was not statistically significant (14.89 vs. 9.41, *p* = 0.215, OR = 1.74).

### 3.2. Preoperative Characteristics

No differences between groups were observed regarding smoking, preoperative BMI, WHO performance status, and the Charlson comorbidity score.

The CCD group presented higher numbers of anemia (22.34 vs. 7.06%, *p* = 0.002) and lower levels of preoperative albumin (3.47 vs. 3.73, *p* < 0.001). Furthermore, a higher percentage of patients had very low albumin levels (below 3 g/dl) in this group (15.59 vs. 2.35, *p* = 0.001).

No differences between groups were observed regarding time from diagnosis to surgery. However, the CCD group had significantly more requirements for emergency procedures (26.60 vs. 8.24%, *p* < 0.001). The most frequent causes for emergency surgery were abdominal abscesses (49.12%) and intestinal obstruction (38.60%).

In the CCD group, 93 patients (50%) presented a stricturing pattern, 64 patients (34%) had a fistulizing pattern, and 30 patients (16%) had both complications. Furthermore, the CCD group presented a history of perianal disease more frequently (17.02 vs. 8.24%, *p* = 0.055).

No differences were observed between groups relating to exposure to perioperative chronic steroids, history of receiving biologic drugs (and number of different lines of biologics), and time from first biological drug received to surgery. Furthermore, both groups had similar exposure to biological drugs within 12 weeks prior to surgery.

Lastly, the CCD group required more in-hospital nutritional optimization before surgery (9.04 vs. 1.18%, *p* = 0.015).

Table 1 summarizes the preoperative information.

### 3.3. Intraoperative Variables

Operating time was significantly shorter in the ECD group (115 vs. 126 min, *p* = 0.012), with a larger percentage of patients in the CCD being subjected to prolonged (more than 150 min) surgical time (17.02 vs. 8.24%, *p* = 0.055, OR = 2.36).

The ECD group had a significantly higher rate of laparoscopic procedures (98.82 vs. 92.55%, *p* = 0.035, OR = 6.72) and a lower conversion rate (2.35 vs. 18.62%, *p* < 0.001, OR = 10.27). The most common causes for conversion in this cohort were the presence of an inflammatory tumor (45%), followed by the invasion of other organs (32%).

On the contrary, CCD had significantly higher requirements for extended resections (right colectomy) and associated surgeries (19.15 vs. 1.18%, *p* < 0.001, OR = 21). Requirements of more than 1 intestinal resection (3.72 vs. 0%, p = 0.055) and intraoperative complications (3.72 vs. 0%, *p* = 0.072) were only found in the CCD group.

The most common associated surgeries in the CCD group were primary repair of the sigmoid colon (36%), primary repair of the small bowel, and enterectomy (20.5%).

Finally, a significantly higher percentage of patients received a primary anastomosis in the ECD group (100 vs. 93.58%, *p*: 0.018), and therefore avoided a stoma.

Intraoperative results are summarized in Table 2.

### 3.4. Postoperative Results

Patients in the CCD group had longer hospitalization (7.40 vs. 5.45 days, *p* = 0.093), with a higher percentage of patients requiring more than one week of hospital stay. Furthermore, a higher number of CCD patients required prolonged ICU admission during the postoperative period (4.26 vs. 0%, *p* = 0.052).

CCD patients also presented a higher postoperative incidence of abdominal abscesses, fascial rupture, and clinically significant gastrointestinal bleeding, although none of these differences were statistically significant. The majority of the patients in the CCD group that presented gastrointestinal bleeding after surgery required a reoperation, whereas none of the ECD patients were re-operated for this reason.

Anastomotic leakage was also numerically more frequent in the CCD group (6.82 vs. 3.53%, *p* = 0.285).

Lastly, reoperations and requirements for re-hospitalization were higher in the CCD group. The mean length of the bowel was similar in both groups (27 vs. 24 cm., *p* = 0.143).

**Figure 3:**
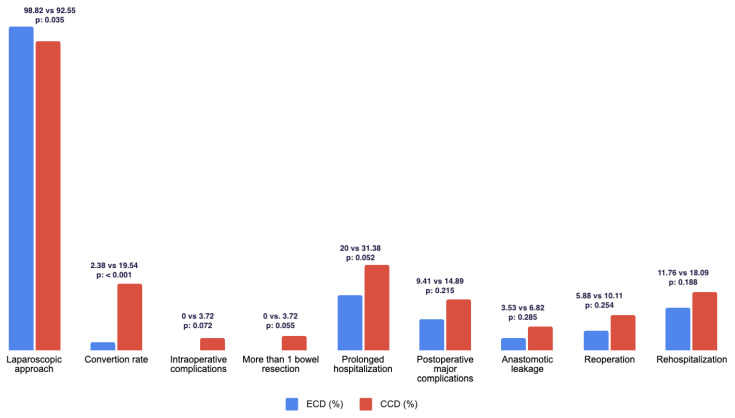
Summary of main operative outcomes for each group.

Postoperative results are summarized in Table 3 and Figure 3.

### 3.5. Multivariate Analysis

In the multivariate analysis, complicated Crohn’s disease was not independently related to any of the main postoperative outcomes (prolonged hospitalization, major postoperative complications, anastomotic leakage, reoperation, and re-hospitalization).

The results of the multivariate analysis are shown in Table 4.

## 4. Discussion

This manuscript is comprised of a number of patients in a single center, each operated on for Crohn’s disease affecting the ileocecal region. The analysis focuses on patients who were operated on while they still presented luminal (inflammatory) disease and compares them with cases operated on for complications of the disease.

A reasonable hypothesis would be that the latter would achieve worse postoperative outcomes.

The first striking fact is the number of patients operated in an early fashion. After the LIRC trial, which suggested that surgery could be considered a reasonable alternative to medical treatment as a second-line strategy [8,9,10], only a few centers seem to have adopted this idea, and therefore, studies showing the outcomes of patients operated on for luminal disease are limited.

Furthermore, according to ECCO guidelines, the first-line treatment after failing conventional medication for these patients should be biologic drugs [19]. Additionally, the most common indication for surgery in Crohn’s patients is for those who present fibrotic strictures or fistulae, since such complications are usually not amenable to medical treatment [13,20,21].

A manuscript presented by the LATAM IBD Surgical Consortium [6] made a similar comparison between complicated and luminal patients operated on at 10 centers in Latin America, the latter representing 17% of the whole cohort. That study showed differences in favor of an early surgical approach for patients: operative time was significantly shorter, with lower rates of intraoperative complications and more primary anastomoses. The complicated group, on the other hand, had non-significant differences in terms of hospital stay (8.3 vs. 6.4 days, *p* = 0.208), and higher rates of postoperative complications (33 vs. 17%, *p* = 0.548) and anastomotic leakage (6.67 vs. 1.67%, *p* = 0.173). Lastly, reoperations were significantly more frequent in the complicated group. (13.36 vs. 3.33%, *p* = 0.026).

The results of the present study are similar to that of the LATAM group, yet differences between the groups are less significant, and some of the overall patient outcomes are better, such as the number of patients operated on using a minimally-invasive approach, conversion rates, and rates of primary anastomosis. There are two plausible explanations for this phenomenon. Firstly, most centers in the LATAM study did not have an active policy to consider surgery for luminal patients, as they mostly operated on severely complicated patients. Secondly, having an active MDT discussion at each step of the way, with an early participation of the surgical team in decision-making along with the rest of the specialists, could be helpful to better decide on the optimal time to perform a surgical resection. Then, even when complications are sustained, the burden of the disease might be lower, as shown in the present cohort.

Some other studies have focused on a different concept of early surgery in patients affected by Crohn’s disease, using a definition based on the time from diagnosis to surgery [22,23]. Furthermore, Kotze et al. [5] presented improved postoperative outcomes in patients who were operated on for Crohn’s disease during the first 5 years from diagnosis, compared to delayed operations.

Nevertheless, CD is a progressive disease as pointed out by Cosnes et al. [11]. Some patients develop disease-related complications after a short duration of the disease, whereas others have an uncomplicated disease for many years. The concept of “early surgery” should then be that surgery is performed before disease-related complications arise, not that it is performed within a certain time limit. Given this definition, early surgery could break from accepted standards of CD management that recommend surgery only for complicated disease.

An example of this can be seen in this study, where time from diagnosis to surgery was very similar in both groups (52 vs. 54 months, *p* = 0.798). Then, the actual benefit of early surgery could be based not on an actual time period between diagnosis and surgery, but rather on an active and continuous discussion of these patients at each stage of treatment, which would help to select the proper timing for surgery.

The aforementioned strategy would also aid in avoiding unnecessary delays in surgical indications for complications of the disease, with patients being subjected to subsequent lines of medical treatment even with a low likelihood of success. This factor (denominated period of clinical deterioration by Iesalnieks et al., referring to prolonged time between the complication, perforating disease, and the indication for surgery) [4] has been deemed to be an independent predictor of worse postoperative outcomes.

This study presents limitations mainly related to the retrospective nature of the design, and the limited number of patients included (which may lead to type II errors, reflected in the broad confidence intervals of the multivariate analysis). Furthermore, the patients were operated on in a hospital located in a country with a strong policy for centralization of care; therefore, results might differ in other populations with different practices.

However, being a single-center study also holds some strengths, namely the systematic practice regarding Crohn’s disease patients, and a solid and experienced MDT to make decisions. This fact, along with the percentage of luminal patients included in this cohort (which proves the strong advocacy of the medical team to consider surgery as a valid alternative to medical treatment in short-segment ileocecal Crohn’s patients), provides value to this manuscript.

Further studies are planned to focus on long-term follow-up of these patients, to assess patterns of relapse and efficacy of medical treatment.

## 5. Conclusions

An early multidisciplinary discussion of patients with CD limited to the ileocecal region is associated with improved operative outcomes, especially in patients intervened with for luminal involvement only. Further studies on this cohort will be directed to assess long-term differences between patients operated for luminal and for complicated disease.

## Figures and Tables

**Table 1:** Preoperative data.

Variables	All Patients *N* = 273 (100%)	ECD *N* = 85 (31.14)	CCD N = 188 (68.86)	*p* Value	Missing Values
Sex, female (n, %)	160 (59.04)	55 (64.71)	105 (55.85)	0.169	0
Age (mean, range)	37.49 (10–79)	38.76 (10–76)	36.92 (10–79)	0.430	1
Smoking	75 (28.25)	27 (32.14)	49 (26.49)	0.340	4
BMI (mean, range)	24.60 (11.4–42.6)	24.73 (11.4–42.6)	24.54 (14–41)	0.784	3
Low BMI (<20)	52 (19.26)	12 (14.29)	40 (21.51)	0.164	
High BMI (>30)	49 (18.15)	14 (16.67)	35 (18.82)	0.671	
WHO performance status >2	6 (2.20)	1 (1.18)	5 (2.66)	0.439	0
Charlson comorbidity score				0.471	0
0–2	227 (83.15)	68 (80)	159 (84.57)		
2–3	35 (12.82)	14 (16.47)	21 (11.17)		
>3	11 (4.03)	3 (3.53)	8 (4.26)		
Anemia	48 (17.58)	6 (7.06)	42 (22.34)	0.002	0
Preoperative albumin (median, range)	3.56 (1.7–4.9)	3.73 (3–4.6)	3.47 (1.7–4.9)	<0.001	0
Low preoperative albumin (<3)	31 (11.44)	2 (2.35)	29 (15.59)	0.001	
Weight loss	79 (28.94)	18 (21.18)	61 (32.45)	0.057	0
Kilos (mean, range)	8.98 (1–30)	9.32 (3.23)	8.87 (1–30)	0.785	5
Previous abdominal surgery	60 (21.98)	16 (18.82)	44 (23.40)	0.397	0
Time from diagnosis to surgery (months, median, range)	52 (0–298)	52 (1–298)	53 (0–284)	0.943	0
More than 2 years	144 (53.14)	40 (47.62)	104 (55.61)	0.222	
More than 5 years	90 (33.21)	28 (33.33)	62 (33.16)	0.255	
Emergency surgical procedure	57 (20.88)	7 (8.24)	50 (26.60)	<0.001	0
Montreal classification					1
A1	20 (7.35)	8 (9.52)	12 (6.38)	0.438	
A2	168 (61.76)	44 (52.38)	124 (65.96)	0.036	
A3	84 (30.51)	32 (38.10)	52 (27.81)	0.090	
B2			93 (35.07)		
B3			95 (34.80)		
Previous perianal disease	39 (14.29)	7 (8.24)	32 (17.02)	0.055	0
Chronic steroids at the time of surgery	39 (14.29)	9 (10.59)	30 (15.96)	0.240	0
Previous exposure to biologics	133 (48.72)	40 (47.06)	93 (49.47)	0.712	0
Number of biologics received before surgery				0.242	0
1	79 (28.78)	22 (26.19)	57 (30.48)	0.486	
2	41 (15.50)	17 (20.24)	24 (13.37)	0.145	
3	10 (3.69)	1 (1.19)	9 (4.81)	0.145	
>3	3 (2.24)	0	3 (1.60)	0.253	
Time from first biologic to surgery (months, mean, range)	39 (1–152)	32 (1–112)	43(1–152)	0.111	5
Previous complications associated to biologics	42 (15.44)	15 (17.65)	27 (14.44)	0.497	1
Exposure to biologics within 12 weeks before surgery	95 (34.80)	26 (30.59)	69 (36.70)	0.282	0
Infliximab	44 (46.32)	15 (57.69)	29 (42.03)		
Adalimumab	38 (40)	9 (34.62)	29 (42.03)		
Vedolizumab	5 (5.26)	1 (3.85)	4 (5.80)		
Ustekinumab	5 (5.26)	1 (3.85)	4 (5.80)		
Other	3 (3.16)	0	3 (4.35)		
Requirement of preoperative nutritional optimization	18 (6.59)	1 (1.18)	17 (9.04)	0.015	0
ASA				0.450	0
I	40 (14.65)	12 (14.12)	28 (14.89)		
II	218 (79.85)	71 (83.53)	147 (78.19)		
III	13 (4.76)	2 (2.35)	11 (5.85)		
IV	2 (0.73)	0	2 (1.06)		

**Table 2:** Operative data.

Variables	All Patients *N* = 273 (100%)	ECD *N* = 85 (31.14)	CCD *N* = 188 (68.86)	*p* Value	OR	Missing Variables
Operating time (median, range)	121 (28–305)	115 (49–286)	126 (57–305)	0.012		0
Prolonged surgery (>150 min)	39 (14.29)	7 (8.24)	32 (17.02)	0.055	2.36	
Laparoscopic approach	258 (94.51)	84 (98.82)	174 (92.55)	0.035	6.72	0
Conversion rate	36 (13.19)	2 (2.35)	34 (18.62)	<0.001	10.27	0
Extension of surgery				0.175	N/A	0
Ileocecal resection	269 (98.53)	85 (100)	184 (97.87)			
Right colectomy	4 (1.47)	0	4 (2.13)			
Mesenteric resection				0.501	N/A	0
Mesenteric sparing	272 (99.63)	85 (100)	187 (99.47)			
Wide mesenteric resection	1 (0.37)	0	1 (0.53)			
Associated surgery	37 (13.55)	1 (1.18)	36 (19.15)	<0.001	21.00	0
Number of patients requiring more than 1 bowel resection	7 (2.56)	0	7 (3.72)	0.055	N/A	0
Intraoperative complications	7 (2.56)	0	7 (3.72)	0.072	N/A	0
CLASSIC Minor	7 (100)	0	7 (100)			
CLASSIC Major	0	0	0			
Primary anastomosis	261 (95.60)	85 (100)	176 (93.62)	0.017	N/A	0
Type of anastomosis				0.597	N/A	0
Hand-sewn	2 (0.77)	1 (1.18)	1 (0.57)			
Stapled	259 (99.23)	84 (98.82)	175 (99.43)			
Anastomosis orientation				0.488	N/A	
End to end	1 (0.38)	0	1 (0.57)			
Side to side	260 (99.62)	85 (100)	175 (99.43)			
Anastomosis orientation				0.281	N/A	
Isoperistaltic	8 (3.07)	5 (5.88)	3 (1.70)			
Antiperistaltic	253 (96.93)	80 (94.12)	173 (98.30)			

**Table 3:** Postoperative data.

Variables	All Patients *N* = 273 (100%)	ECD *N* = 85 (31.14)	CCD *N* = 188 (68.86)	*p* Value	OR	Missing Variables
Hospitalization days (mean, range)	6.80 (1–96)	5.45 (1–39)	7.40 (2–96)	0.093		0
Prolonged hospitalization (more than 7 days)	76 (27.84)	17 (20)	59 (31.38)	0.052	1.83	
Requirement of prolonged postoperative ICU	8 (2.93)	0	8 (4.26)	0.054	N/A	0
Postoperative major complications	36 (13.19)	8 (9.41)	28 (14.89)	0.215	1.74	0
Abdominal abscess	15 (5.49)	3 (3.53)	12 (6.38)	0.348	1.84	0
Fascial rupture	7 (2.56)	1 (1.33)	6 (3.19)	0.489	2.74	0
Postoperative gastrointestinal bleeding	7 (2.56)	1 (1.19)	6 (3.21)	0.441	2.74	0
Reoperation for bleeding	4 (57.14)	0	4 (66.67)			
Surgical site infection	5 (1.85)	1 (1.19)	4 (2.14)	0.592		0
Anastomotic leakage	15 (5.75)	3 (3.53)	12 (6.82)	0.285	2.00	0
Minor leakage	4 (26.67)	1 (33.33)	3 (25)			
Major leakage	11 (73.33)	2 (66.67)	9 (75)			
Readmission	44 (16.12)	10 (11.76)	34 (18.09)	0.188	1.69	0
Reoperation	24 (8.79)	5 (5.88)	19 (10.11)	0.254	1.88	0
Mortality	1 (0.37)	0	1 (0.53)	0.501	N/A	0
Length of resected bowel (cm, mean, range)	26 (5–158)	24 (7–140)	27 (5–158)	0.143		4

**Table 4:** Multivariate analysis considering main operative outcomes as dependent variables.

Variable	OR	Standard Error	*p* Value	95% CI
Prolonged hospitalization (>1 week)	2.69	2.24	0.24	0.53–13.71
Major postoperative complications	0.77	0.57	0.72	0.18–3.33
Anastomotic leakage	1.14	1.18	0.90	0.15–8.74
Reoperation	1.86	1.80	0.52	0.28–12.51
Re-hospitalization	2.65	1.62	0.11	0.80–8.80

## Data Availability

Data available upon reasonable request.

## Appendix A. Algorithm of Management Used at Our Institution for Management of Patient with Ileocecal CD

At our institution, high-resolution entero-MRI informed by a highly trained specialist is a very important tool used to differentiate between luminal affection only from patients who have a fibrotic stenosis (that could be an absolute indication for surgery). This is possible due to the ability of MRI to visualize the entire intestinal wall.

The institutional protocol includes all of the necessary sequences previously described for correctly assessing activity in Crohn’s disease [24,25,26].

To sum up, the strongest indicators of inflammation are intramural edema, which is seen as hyperintensity on T2-weighted fat-saturated images. Other indicators are homogenous wall enhancement, mural thickness, mural ulcers, Comb sign (see Figure A2), and restricted diffusion on diffusion-weighted sequences.

Below there is an image of an ileocecal CD patient, showing a coronal contrast enhanced T1-weighted MR enteroclysis with biphasic oral contrast. This shows bowel wall thickening, hypervascularity, and Comb sign (engorgement of the mesenteric vessels with vascular dilatation, tortuosity with spacing of the vasa recta), indicating inflammation/luminal CD.

## References

[1] Thoreson R., Cullen J. (2007). Pathophysiology of inflammatory bowel disease: An overview. Surg. Clin. N. Am..

[2] Gomollón F., Dignass A., Annese V., Tilg H., Van Assche G., Lindsay J.O., Peyrin-Biroulet L., Cullen G.J., Daperno M., Kucharzik T. (2017). 3rd European evidence-based consensus on the diagnosis and management of Crohn’s disease 2016: Part 1: Diagnosis and medical management. J. Crohn’s Colitis.

[3] Yamamoto T., Lightner A.L., Spinelli A., Kotze P.G. (2020). Perioperative management of ileocecal Crohn’s disease in the current era. Expert Rev. Gastroenterol. Hepatol..

[4] Iesalnieks I., Kilger A., Glaß H., Obermeier F., Agha A., Schlitt H.J. (2010). Perforating Crohn’s ileitis: Delay of surgery is associated with inferior postoperative outcome. Inflamm. Bowel Dis..

[5] Kotze P., Magro D.O., Martinez C.A.R., Spinelli A., Yamamoto T., Warusavitarne J., Coy C.S.R. (2018). Long Time from Diagnosis to Surgery May Increase Postoperative Complication Rates in Elective CD Intestinal Resections: An Observational Study. Gastroenterol. Res. Pract..

[6] Surgical IBD LATAM Consortium (2022). Earlier surgery is associated to reduced postoperative morbidity in ileocaecal Crohn’s disease: Results from SURGICROHN—LATAM study. Dig. Liver Dis..

[7] Sebastian S., Segal J.P., Hedin C., Pellino G., Kotze P.G., Adamina M., Campmans-Kuijpers M., Davies J., de Vries A.C., Casbas A.G. (2022). ECCO Topical Review: Roadmap to optimal peri-operative care in IBD. J. Crohn’s Colitis.

[8] Ponsioen C.Y., de Groof E.J., Eshuis E.J., Gardenbroek T.J., Bossuyt P.M., Hart A., Warusavitarne J., Buskens C.J., van Bodegraven A.A., Brink M.A. (2017). Laparoscopic ileocaecal resection versus infliximab for terminal ileitis in Crohn’s disease: A randomized controlled, open-label, multicentre trial. Lancet.

[9] De Groof E.J., Stevens T.W., Eshuis E.J., Gardenbroek T.J., Bosmans J.E., van Dongen J.M., Mol B., Buskens C.J., Stokkers P.C., Hart A. (2019). Cost-effectiveness of laparoscopic ileocaecal resection versus infliximab treatment of terminal ileitis in Crohn’s disease: The LIR!C Trial. Gut.

[10] Stevens T.W., Haasnoot M.L., D’Haens G.R., Buskens C.J., de Groof E.J., Eshuis E.J., Gardenbroek T.J., Mol B., Stokkers P.C.F., Bemelman W.A. (2020). Laparoscopic ileocaecal resection versus infliximab for terminal ileitis in Crohn’s disease: Retrospective long-term follow-up of the LIR!C trial. Lancet Gastroenterol. Hepatol..

[11] Cosnes J., Cattan S., Blain A., Beaugerie L., Carbonnel F., Parc R., Gendre J.P. (2002). Long-term evolution of disease behavior of Crohn’s disease. Inflamm. Bowel Dis..

[12] Pellino G., Sampietro G.M. (2023). Defining the role of abdominal surgery and its impact on the disease course in patients with Crohn’s disease: Unsolved issues and novel insights. Dig. Liver Dis..

[13] Maruyama B.Y., Ma C., Panaccione R., Kotze P.G. (2022). Early Laparoscopic Ileal Resection for Localized Ileocecal Crohn’s Disease: Hard Sell or a Revolutionary New Norm?. Inflamm. Intest. Dis..

[14] Bemelman W.A., Warusavitarne J., Sampietro G.M., Serclova Z., Zmora O., Luglio G., Overstraeten A.D.B.V., Burke J.P., Buskens C.J., Francesco C. (2017). ECCO-ESCP consensus on surgery for Crohn’s Disease. J. Crohn’s Colitis.

[15] Rosenthal R., Hoffmann H., Clavien P.A., Bucher H.C., Dell-Kuster S. (2015). Definition and Classification of Intraoperative Complications (CLASSIC): Delphi Study and Pilot Evaluation. World J. Surg..

[16] Coffey C.J., Kiernan M.G., Sahebally S.M., Jarrar A., Burke J.P., Kiely P.A., Shen B., Waldron D., Peirce C., Moloney M. (2018). Inclusion of the mesentery in ileocolic resection for Crohn’s disease is associated with reduced surgical recurrence. J. Crohn’s Colitis.

[17] Dindo D., Demartines N., Clavien P.A. (2004). Classification of surgical complications: A new proposal with evaluation in a cohort of 6336 patients and results of a survey. Ann. Surg..

[18] Rahbari N.N., Weitz J., Hohenberger W., Heald R.J., Moran B., Ulrich A., Holm T., Wong W.D., Tiret E., Moriya Y. (2010). Definition and grading of anastomotic leakage following anterior resection of the rectum: A proposal by the International Study Group of Rectal Cancer. Surgery.

[19] Torres J., Bonovas S., Doherty G., Kucharzik T., Gisbert J.P., Raine T., Adamina M., Armuzzi A., Bachmann O., Bager P. (2019). ECCO Guidelines on Therapeutics in Crohn’s Disease: Medical Treatment. J. Crohn’s Colitis.

[20] Pellino G., Siccr T.I.S.O.C.S., Keller D.S., Sampietro G.M., Angriman I., Carvello M., Celentano V., Colombo F., Di Candido F., Laureti S. (2020). Italian Society of Colorectal Surgery SICCR. Inflammatory bowel disease position statement of the Italian Society of Colorectal Surgery (SICCR): Crohn’s disease. Tech. Coloproctology.

[21] Pellino G., Keller D.S., Sampietro G.M., Annese V., Carvello M., Celentano V., Coco C., Colombo F., Cracco N., di Candido F. (2020). The Italian Society of Colorectal Surgery (SICCR). Inflammatory bowel disease (IBD) position statement of the Italian Society of Colorectal Surgery (SICCR): General principles of IBD management. Tech. Coloproctology.

[22] Sarikaya M., Zhao M., Lo B., Bendtsen F., Burisch J. (2022). Disease course and treatment outcomes of Crohn’s disease patients with early or late surgery—A Danish nationwide cohort study from 1997 to 2015. Dig. Liver Dis..

[23] An V., Cohen L., Lawrence M., Thomas M., Andrews J., Moore J. (2016). Early surgery in Crohn’s disease a benefit in selected cases. World J. Gastrointest. Surg..

[24] Punwani S., Rodriguez-Justo M., Bainbridge A., Greenhalgh R., De Vita E., Bloom S., Cohen R., Windsor A., Obichere A., Hansmann A. (2009). Mural inflammation in Crohn disease: Location-matched histologic validation of MR imaging features. Radiology.

[25] Zappa M., Stefanescu C., Cazals-Hatem D., Bretagnol F., Deschamps L., Attar A., Larroque B., Tréton X., Panis Y., Vilgrain V. (2011). Which magnetic resonance imaging findings accurately evaluate inflammation in small bowel Crohn’s disease? A retrospective comparison with surgical pathologic analysis. Inflamm. Bowel Dis..

[26] Bruining D.H., Zimmermann E.M., Loftus E.V., Sandborn W.J., Sauer C.G., Strong S.A. (2018). Consensus Recommendations for Evaluation, Interpretation, and Utilization of Computed Tomography and Magnetic Resonance Enterography in Patients With Small Bowel Crohn’s Disease. Gastroenterology.

